# *Bacillus toyonensis* SAU-19 and SAU-20 Isolated From *Ageratina adenophora* Alleviates the Intestinal Structure and Integrity Damage Associated With Gut Dysbiosis in Mice Fed High Fat Diet

**DOI:** 10.3389/fmicb.2022.820236

**Published:** 2022-02-17

**Authors:** Samuel Kumi Okyere, Juan Wen, Yujing Cui, Lei Xie, Pei Gao, Ming Zhang, Jianchen Wang, Shu Wang, Yinan Ran, Zhihua Ren, Yanchun Hu

**Affiliations:** ^1^Key Laboratory of Animal Disease and Human Health of Sichuan Province, College of Veterinary Medicine, Sichuan Agricultural University, Chengdu, China; ^2^New Ruipeng Pet Healthcare Group Co., Ltd., Shenzhen, China

**Keywords:** *Ageratina adenophora*, endophytes, *Bacillus toyonensis* SAU-19, *Bacillus toyonensis* SAU-20, intestinal integrity, tight junction proteins, inflammation cytokines

## Abstract

This study was performed to identify potential probiotic endophytes from *Ageratina adenophora* and evaluate their ameliorating effects on gut injury and integrity damage associated with microbiota dysbiosis in mice fed high fat diet. Using morphological and biochemical tests, and 16S rRNA gene sequencing technique, two bacteria endophytes were identified as strains of *Bacillus toyonensis* and were named *Bacillus toyonensis* SAU-19 (GenBank No. MW287198) and *Bacillus toyonensis* SAU-20 (GenBank No. MW287199). Sixty (60) mice were divided into five groups, group 1 was the negative control fed normal diet (NS), group 2 was fed High fat diet (HF), Group 3 was fed High fat diet + 10^6^ *Lactobacillus rhamnosus* (LGG), group 4 was fed High fat + 10^6^ *Bacillus toyonensis* SAU-19 and group 5 fed High fat diet + 10^6^ *Bacillus toyonensis* SAU-20. After 35 days, histological and immunohistochemistry examination were performed in the ileum tissues. Furthermore, DAO and antioxidants activities were measured in serum, mRNA expressions of tight junction proteins (occludin and ZO-1) and inflammation related cytokines (IL-1β, TFN-α, IL-2, IL-4, and IL-10) in the ileum tissues as well as sIgA levels and total bacteria (*Escherichia coli*, *Salmonella*, *Staphylococcus*, and *Lactobacillus*) in the small intestine and cecum content. The results showed an increase in the DAO activity, oxidative stress parameter (MDA), pro-inflammation cytokines (IL-1β, TFN-α, IL-2), reduce immunity (sIgA), and destroyed intestinal structure and integrity (reduce tight junction proteins) in the high fat diet group and this was associated with destruction of the gut microbiota composition (increasing pathogenic bacteria; *E. coli*, *Salmonella*, *Staphylococcus* and reducing beneficial bacteria, *Lactobacillus* spp.) in mice (*P* < 0.05). However, the administration of *Bacillus toyonensis* SAU-19 and SAU-20 reverted these effects. Our findings indicated that, *Bacillus toyonensis* SAU-19 and SAU-20 isolated from *A. adenophora* could prevent the excess weight gain from high fat diet feeding, improved antioxidant status and alleviated the intestine integrity damage as well as reduce the population of enteric bacteria such as *E. coli, Salmonella*, and *S. aureus* and increasing the population of beneficial bacteria such as *Lactobacillus* in the gut of mice fed high fat diet, therefore, can serve as a potential probiotics in humans and animals.

## Introduction

Probiotics are live microorganisms that, when administered in adequate amounts, confer health benefits on the host (FAO/WHO, 2002). Probiotics competitively occupy the receptors on mucosal epithelial cells and inhibit the adhesion of pathogens to epithelial walls (Miljkovic et al., 2015). Probiotics have been reported to improve intestinal structure, integrity and microbiota (Wang et al., 2020; Yu et al., 2020; Sudan et al., 2021). Over the past years, probiotics were mainly isolated from milk sources, however with the high demand for non-animal probiotic products by vegetarians, isolation and development of probiotics from plant sources is on the rise (Nematollahi et al., 2016).

The internal tissues of plants harbor a class of beneficial endosymbiotic microorgan-isms (predominantly bacteria and fungi) called endophytes (Kumara et al., 2014). In this plant-endophyte relationship, plants offer nourishment and protection to the endophytes, whereas, endophytes enhance defense, health, and stress tolerance of plants (Kandel et al., 2017). Numerous studies have isolated several endophytes with probiotics potential from fruits, leaves and other plant parts (Touret et al., 2018; Pabari et al., 2020; Ragul et al., 2020; Unban et al., 2020).

The *Asteraceae* plant, *Ageratina adenophora* (Spreng) R. M. King et H. Rob. is the most destructive invasive plant that threatens the native biodiversity in the southwestern regions of China (Sun et al., 2018). In addition, studies have indicated that, this plant induces toxicity in both humans and animals (Ouyang et al., 2014; He et al., 2016; Mo et al., 2017; Okyere et al., 2020; Ren et al., 2021a,b). Currently, research on *A. adenophora* has gradually moved from toxicity prevention to biological utilization of the plant *in vivo* (Xu et al., 2018; Chen L. et al., 2020). Due to the invasive nature and growth performance of this plant, some scientists are focusing on harnessing the chemical substances and other products in the plant’s resources and developing them into useful products (Fu et al., 2019; Okyere et al., 2021). Therefore, we hypothesized that, *A. adenophora* may contain endophytes that have potential probiotic activity.

High fat diets cause a shift in gut bacterial community and a modest increase in circulating endotoxin concentrations (Cani et al., 2007). The dysbiosis of the gut microbiota may results in disturbances in the normal physiological processes of the body, hence, may affect the overall mice growth performance and gut integrity via increasing the population of harmful bacteria and viruses which invade tissues and produce lethal toxins and metabolites to damage them (Abaidullah et al., 2019).

Thus, this study was performed for the first time to isolate, characterize and identify potential probiotic endophytes from *A. adenophora* plant and evaluate its ameliorative effects (*in vivo*) on intestinal structure and integrity damage, and gut microbiota dysbiosis induce by high fat diet in mice. This study will give the basis for further investigation and future development of probiotics and identification of other beneficial endophytes from *A. adenophora*, thereby utilizing the plant resource for human and animal benefit.

## Materials and Methods

### Sample Collection

Leaves and stems of *A. adenophora* were collected from Wangsuo Village, Cangzhou Street, Dechang County, Liangshan Yi Autonomous Prefecture, Sichuan Province (102°15′20′′ E and 27°20′11′′ N; elevation = 2152 m). The samples were confirmed as *A. adenophora* by Prof. Chao Hu, Department of Botany, Sichuan Agricultural University. Culture media were purchased from Qingdao Hope Bio-Technology Co., Ltd., Qingdao, China. Mice, basal and high fat diet (Table 1) were purchased from Chengdu Dashuo Experiment Animal Co. Ltd., Chengdu, China. *E. coli* CMCC44103, *Salmonella typhimurium* ST19, and *Lactobacillus rhamnosus* GG ATCC 53103 was obtained from the College of Veterinary Medicine (Professor Xueqin Ni’s lab), Sichuan Agricultural University, China.

**TABLE 1:** Composition of the basal diet.

Normal diet		High fat diet	
Ingredients	Content g/kg	Ingredients	Content g/kg
Water	94	Water	93
Protein	190	Protein	134
Fat	51	Fat	143
Fiber	36	Fiber	27
Ash	62	Ash	44
Calcium	11.3	Calcium	8.3
Phosphorus	8.6	Phosphorus	7.1

### Plant Samples Sterilization

Leaf and stem were separated from the plant and washed thoroughly under running tap water to remove adhering soil particles and microbes. The surface decontamination method described in a previous study was used with some minor modification (Fisher et al., 1992). Leaf and stem samples were further cleaned with detergent (5% Teepol), under running tap water and then distilled water. The cleaned samples were then placed in a laminar hood and immersed in 70% ethanol and sodium hypochlorite (5%) solution for 30 s and 3 min respectively. The samples were further exposed to absolute alcohol for 10 s, thereafter; they were washed with autoclaved distilled water three times. Surface decontamination was performed on the samples after cutting them into pieces (1 cm^2^) with sterile surgical blade.

### Isolation and Purification of Potential Probiotic Endophyte

The isolation of potential probiotic endophytes was conducted following the procedures described in a previous study (Bhore et al., 2010). Well grown colonies of cultivable bacterial putative-endophytes that were visible on the LB Agar were randomly selected for further experiments. Pure cultures of each putative endophyte were cultivated separately in universal bottles containing 10 mL LB broth. Culture-cultivation was carried out at 37°C for 20 h. Glycerol stocks (30%) were prepared and kept at –80°C to preserve the putative probiotic endophytes for subsequent research.

### Preliminary Identification of Potential Probiotic Endophyte

Distinctive colonies on LB agar plates were subjected to preliminary identification using colony characteristics, Gram staining and catalases activity.

Colony characteristics such as color, elevation, margins and surfaces of each putative probiotic endophyte were studied using magnifying lens.

#### Gram Staining

Gram staining of the isolates was performed using commercial kits (Nanjing Duly Biotech Co., Ltd., Jiangsu, China) following the manufacturer’s instructions.

#### Catalase Test

Pure colonies of isolates were placed on a slide, and then a drop of hydrogen peroxide (3%) was added to the isolates on the slides. The formation of bubbles showed a catalase positive result, whereas absence of bubbles showed a catalase negative reaction.

Only colonies showing phenotypic features similar to lactic acid bacteria such as gram-positive, catalase-negative reaction, and white or creamy colony color were selected for further studies. We further performed a justification experiment to substantiate the probiotic potential of endophytes through other morphological, biochemical and probiotic tests. These tests included bacteria size, shape and arrangement determination, NaCl, acid and bile salt tolerance, glucose fermentation, antimicrobial activity and antibiotic sensitivity. All tests were performed in triplicate.

### Morphological and Biochemical Characterization of Potential Probiotic Endophyte

The size, shape and arrangement of each putative probiotic endophyte were observed using an electronic microscope (IN-B129 USB Digital Electronic Operation Microscope Driver, Guangzhou, China) (Ahmad and Zargar, 2017).

The isolates were biochemically characterized using glucose, sucrose and D-sorbitol fermenting test, and sodium chloride (NaCl) inhibition test (Zaved et al., 2008).

### Carbohydrate Fermentation Test

Glucose, sucrose and D-sorbitol fermentation were conducted in LB fermentation broth with 0.004% phenol red containing 2% of glucose, sucrose, and D-sorbitol. All the tubes were sterilized for 15 min at 121°C. The tubes were inoculated with a single colony of bacteria. The positive reaction of the bacteria was indicated by the changes in the color of the medium (from red to yellow).

### Tolerance to Inhibitory Substances Test

To observe the NaCl tolerance of the isolates, 1% fresh overnight culture of the isolates was incubated in LB broth with 0, 2, 4, 6.5% NaCl concentration for 24 h. The turbidity in broth was observed as NaCl tolerance (Chakraborty and Bhowal, 2015). Broth with no bile salt was used as a positive control whereas broth with no bile salt and isolate was used as the negative control.

### Probiotic Characterization of Potential Probiotic Endophyte

#### Optimization of Growth Parameters (pH)

LB broths with different pH’s were prepared with 1M HCL and 1M NaOH. The isolated endophyte cultures were inoculated into sterile LB broth tubes of varying pH (i.e., pH 2.5, 4.5, and 6.5) and incubated at 37°C for 24 h. The turbidity of the medium was observed to be pH tolerant (Tambekar and Bhutada, 2010).

#### Bile Salt Tolerance

For the bile salt tolerance test, LB medium with varying concentrations (0, 0.2, and 0.4%) of bile salt [sodium deoxycholate (DCA)] were inoculated with selected endophyte cultures and incubated at 37°C for 24 h. The turbidity of the medium was observed to be bile salt tolerance (Tambekar and Bhutada, 2010). Broth with no bile salt was used as positive control whereas broth with no bile salt and isolate was designated as the negative control.

### Antibiotic Sensitivity Test

Antibiotic sensitivity patterns were evaluated using the commercial kit method (Oxoid Thermo Fisher Biochemicals Ltd., Beijing, China). Different types of common antibiotic disks were loaded onto the surface of the medium and kept at room temperature for 10 min. After that, the plates were incubated at 37°C for 24 h. The diameters of the zones of inhibition were measured in millimeters using a Vernier caliper. The antibiotics tested were tetracycline (30 μg), erythromycin (15 μg), sultamicillin (10 μg), ampicillin (20 μg), and streptomycin (10 μg). Disks immersed in sterile water were used as the negative controls.

Inhibition zones were characterized based on inhibition diameter as resistant (R) (zone < 10 mm), moderately susceptible (MS) (zone 10–20 mm), susceptible (S) (zone > 20 mm), and very susceptible (VS) (zone > 31 mm) (Talib et al., 2019).

### Antimicrobial Activity

The tested organisms *E. coli* CMCC44103 and *Salmonella typhimurium* ST19 were maintained in their respective broths in screw-capped tubes and kept at 4°C. The tested microorganisms were standardized using 0.5 McFarland standards (cell density of 1.5 × 10^8^CFU/mL, with absorbance of 0.132 at a wavelength of 600 nm) (Talib et al., 2019).

For screening of endophyte cultures, these cultures were inoculated in LB broth and incubated at 37°C for 24 h on a shaker to carry out the fermentation process. After incubation, 1 mL of each fermented culture broth was used to test the antimicrobial activity against the two pathogenic bacteria by agar well diffusion method. The endophyte culture was screened against *E. coli* CMCC44103 and *Salmonella typhimurium* ST19. Overnight cultures of pathogens grown in their respective medium at 37°C were diluted to a turbidity equivalent to that of a 0.5 McFarland standard (Khunajakr et al., 2008). The inoculum was spread over the surface of petri dishes containing LB agar (Difco); and then wells were perforated in the agar in which culture supernatants of the selected endophyte was added. Sterile water was used as the control. The plates were then incubated for 24 h at 37°C in a 5% CO_2_ atmosphere. After incubation, the presence of inhibition halos with diameters > 1 mm were considered as positive results (Pessoa et al., 2017).

### 16S rRNA Gene Sequencing of Selected Potential Probiotic Endophyte

The DNA’s of the selected endophytes were extracted using the commercial kit method (QlAamp DNA mini kit-Qiagen). The 16S rRNA was amplified by PCR (Applied Biosystems-9700) using universal primers (27F AGA GTT TGA TCM TGG CTC AG and 1492R GTATTA CCGCGGCTG CTG G) and sequenced using a Genetic Analyzer 3130 (Applied Biosystems, Wakefield, United States) in Solgent Co. Ltd. (16S rRNA, Seoul, South Korea). The aligned 16S rRNA of the isolates were subjected to BLAST using the non-redundant database of NCBI Genbank. Based on the maximum identity score (99% and above) they were selected and aligned using Clustal W multiple alignment software. Considering the results of sequence analyses, a phylogenetic tree was formed via the neighbor-joining method using the software package MEGA X (Jinglong et al., 2019).

### Animal (*in vivo*) Experiment

#### Experimental Animals

A total number of 72 (5 weeks old; BW 25–30 g) live and apparently healthy male Specific-pathogen free) Kun ming mice (SPFKM) mice were used in this study. Twelve (12) mice were used for the pilot experiment, and 60 mice were used in the feeding experiment. For the pilot experiment, mice were allocated equally into three groups; the contro1 group was fed normal diet and 1 ml of 0.9% normal saline in drinking water while the other groups were fed normal diet and 1 ml of 1 × 10^6^ (minimum probiotic consumption range) (Kechagia et al., 2013) of selected endophyte suspension in drinking water for 20 days at a controlled temperature (range 22–24°C) and humidity (range 40–60%) room. Mortality, illness (such as diarrhea) and feed intake were recorded each day until the day 20. The results indicated that daily administration of both endophytes did not reduce feed intake or cause any illness and mortality, hence it was safe for use in the experiment. This study was approved by the Institutional Animal Care and Use Committee of Sichuan Agricultural University, Sichuan, China, under the permit number DKY-B2019603005. The study was also performed in accordance with the ARRIVE guidelines^1^.

### Commercial Probiotic

*Lactobacillus rhamnosus* GG ATCC 53103 is a commercial product being used all over the world. It was originally isolated from feces of a healthy human adult by Professor Xueqin Ni’s lab in College of Veterinary Medicine, Sichuan Agricultural University, Chengdu, China. This bacterial strain improved the growth and health of poultry and large animals at a safe dose range of 1 × 10^6^ –1 × 10^11^ CFU/ml or CFU/g. In addition, the beneficial effects of this strain have been widely studied in both clinical and human intervention studies (Segers and Lebeer, 2014), hence we selected and used it as a bench mark for comparing our isolates effectiveness and safety in this study. We obtained pure samples and made fresh samples for administration each week and stored them in a refrigerator (4°C) until the 35 days of the experiment were finished.

### Experimental Design

Sixty (60) male mice (5 weeks old; BW 25–30 g) were randomly allocated into five groups; 12 mice per group, and 4 mice per replicate in each group. Group A (negative control) was fed a normal diet (20 g normal diet) + 1mL daily amounts of 0.9% normal saline (NC), Group B (positive control) was fed a High fat diet (20 g) + 1mL daily amounts of 0.9% normal saline (HF), Group C was fed High fat diet (20 g) + 1 mL daily of 1 × 10^6^ CFU mL^–1^ *Lactobacillus rhamnosus* GG suspension (HF + LGG), Group C was fed a High fat diet (20 g) + 1 mL of 1 × 10^6^ CFU mL^–1^ *Bacillus toyonensis* SAU-19 (HF + *B. toyo. SAU-19*) and Group D fed with High fat diet (20 g) + 1 mL of 1 × 10^6^CFU mL^–1^ *Bacillus toyonensis* SAU-20 (HF + *B. toyo. SAU-20*) suspension in drinking water for 35 days. Experimental duration was selected according to our previous work (unpublished) which showed that 35 days of feeding of high fat diet could cause gut dysbiosis and the work by Delgado et al. (2009) and Xin et al. (2020). The administration of both the high fat diet and putative probiotics were initiated the same time in this study. The mice were housed in cages at the departmental experimental house and were exposed to a 12 h cycle of light and darkness. The experiment was carried out in a temperature (range 22–24°C) and humidity (40–60%) controlled environment. The animals were monitored daily for any abnormality, illness (such as diarrhea) and death. Throughout the experiment, at exactly 8:00am, normal (NS), high fat diet, high fat diet + commercial probiotic (NP) and high fat diet + selected potential probiotic endophytes were supplemented to their respective groups. Feed intake was monitored and recorded daily throughout the experimental period. Clean water was provided *ad libitum* in clean water bottles. Water bottles were washed every week and fresh drinking water was placed in it for the next week’s administration. The bedding material (wood shavings) was also changed weekly. To administer the potential probiotic endophyte and LGG, new stocks were generated each week in LB and MRS broths and their viability was monitored by serial dilution and viable cell count using LB and MRS agar respectively.

After the 35-day experimental period, the general health, weight gain, and feed conversion ratio were measured. Subsets of the mice were euthanized by CO_2_ asphyxiation on (*n* = 8 mice/group); all mice were fasted for 12 h prior to being euthanized. At necropsy, blood from the abdominal aorta was collected into anticoagulant-coated tubes; and the small intestine was then aseptically removed and processed as indicated in the assays outlined below.

### Preparation of Bacterial Suspension

Potential probiotic endophytes were transferred twice into LB broths and incubated anaerobically at 37°C for 72 h. The bacterial cells were collected by centrifugation (3,500 g, 5 min), and washed twice in 0.85% NaCl (Sigma), and then resuspended in 0.85% NaCl to a final concentration of 1 × 10^6^ CFU/mL and stored at 4°C.

### Histopathological Analysis

After weighing the isolated intestine, the ileum section was cut aseptically and samples (1/2 of each intestine collected at necropsy) were fixed in 10% buffered neutral formalin solution, and then dehydrated in graded (70–100%) alcohol. After being cleared in xylene, each sample was embedded in paraffin and 5 μm-thick sections were prepared. For analyses, samples were stained with hematoxylin and eosin (H&E) dye and then evaluated using a light microscope. A total of eight mice per group were evaluated; for each mouse, four stained slices were examined. Two independent investigators who were blinded to the treatment evaluated the slides. A 0–3 point scale was used to describe the severity of inflammation (0 = none, 1 = mild, 2 = moderate, and 3 = severe). Each parameter was calculated and summed to obtain the overall score (Jang et al., 2019; Fan et al., 2021). Slides were also blinded, and Image Pro Plus 6.0 (Media Cybernetics, Bethesda, MD, United States) software was used to measure villus height and crypt depth. A villus was considered suitable to measure if it was completely intact and had two intact crypts on both sides. Suitable crypts were those that were intact and flanked by two intact villi.

### Determination of Diamine Oxidase Levels in Serum of Mice

The concentration of diamine oxidase (DAO) was determined in the blood serum using commercial ELISA kits (Jingmei Biological Technology, Jiangsu, China) to assess the degree of gut mucosal barrier damage, according to the manufacturer’s instructions. The level of sensitivity of each kit was 0.1 pg/ml.

### Determination of Antioxidant Capacity in Serum of Mice

Antioxidant indexes in serum at 35 days were measured using commercial kits (Nanjing Jiancheng Bioengineering Institute, Nanjing, Jiangsu, China) following the manufacturer’s instructions. The antioxidant parameters checked were catalase (CAT), superoxide dismutase (SOD), glutathione (GSH), and malondialdehyde (MDA).

### Determination of Secretory Immunoglobulin A Levels in Small Intestine Contents of Mice

Fresh small intestine contents of two mice from each replicate were sampled in 5-ml microcentrifuge tubes at day 35 and stored at –80°C. ELISA kits (Sagon Biotech, Shanghai, China) were used to enumerate Secretory Immunoglobulin A (sIgA) levels in the small intestine contents following the manufacturer’s instructions. The level of sensitivity of the kit was 0.1 μg/ml.

### Immunohistochemistry Assay

The small intestine was carefully dissected from the mice, washed with cold PBS (pH 7.2–7.4) and then fixed overnight in 4% paraformaldehyde, and embedded in paraffin wax after dehydration. The paraffin-embedded intestinal tissues were sliced into 4 μm sections and then dewaxed in xylene, followed by rehydrating through a graded series of ethanol solutions. Endogenous peroxidase was blocked by incubating with 3% H_2_O_2_ in methanol for 10 min. After, heat-induced antigen retrieval was performed using citrate buffer (10 mM, pH = 6.0). The sections were blocked with goat serum (Sigma, St. Louis, MO, United States) for 15mins, and incubated overnight at 4°C with a rabbit monoclonal anti-mouse occludin antibody (ab216327, Abcom, dilution 1:200). Each section was rewarmed at 37°C for 1 h and washed in PBS for 10 min, followed by incubation with biotin-tagged anti-rabbit second antibody (SP9001, ZSGB-BIO, Beijing, China) at 25°C for 15mins. Furthermore, horseradish peroxidase-labeled streptavidin working solution (S-A/HRP) was added and after 15 min, the mixture was slowly flushed with PBS. Finally, freshly prepared DAB chromogenic solution was added, then after 6 min, hematoxylin was also added for 20 s. The slides were examined for positive staining and were subjected to optical density analysis with a Leica DM-1000 microscope (Leica Microsystems Imaging Solutions Ltd., Cambridge, United Kingdom) using Image Pro Plus 6.0 (Media Cybernetics, Bethesda, MD, United States), three visual fields were randomly selected in each sections.

### Extraction of Total RNA and Quantitative Real Time Polymerase Chain Reaction

Samples of small intestine tissues (30 mg/mouse) were snap-frozen with liquid N_2_ and then immediately ground into powder using a ceramic mortar. Total RNA from each sample was extracted using an Animal Total RNA Isolation Kit (Sagon Biotech, Shanghai, China) according to manufacturer’s instructions. After confirming the isolated RNA concentration and purity using a NanoDrop One system (Thermo Fisher Scientific, Waltham, MA, United States; OD260/280 ≈ 1.9–2.0), triplicate aliquots (each 1 μg) were removed, loaded into wells, and cDNA was prepared using a PrimeScrip RT reagent kit (Takara, Tokyo, Japan). Thereafter, quantitative real time polymerase chain reaction (qRT-PCR) was performed using a SYBR Premix ExTaq (Takara, Tokyo, Japan) and a CFX96 thermal cycler (Bio-Rad, Hercules, CA, United States). The PCR conditions were shown as follows: 95°C for 5 min, followed by 40 cycles of 95°C, 15 s for denaturation, 60°C, 60 s for annealing at and 70°C, 25 s for extension. Each qRT-PCR reaction was performed with volumes of 10 μL containing 5 μL TB Green TM Premix (Takara), 1 μL forward and reverse primers, 1 μL cDNA, and 2 μL DNase/RNase-Free Deionized Water (Tiangen, Beijing, China). The primers used to analyze the genes of interest were designed from NCBI genBank and are shown in Table 2. Relative gene expression in each sample was normalized to an internal control (β-actin); data analysis was performed using the 2^–ΔΔ^ ^Ct^ method. All samples were evaluated in triplicate.

**TABLE 2:** Primers used for the real-time PCR analysis.

Gene name	Primer	Sequence (5′ and 3′)	Product length (bp)	Annealing Temperature (°C)	Sequence number
IL-1β	Forward	TGAAATGCCACCTTTGACAGTG	141	60.18	NM_008361.4
	Reverse	ATGTGCTGCTGCGAGATTTG			
IL-2	Forward	AGATGAACTTGGACCTCTGCG	175	60.07	NM_008366.3
	Reverse	AAAGTCCACCACAGTTGCTG			
IL-4	Forward	GTACCAGGAGCCATATCCACG	130	60.18	NM_021283.2
	Reverse	TTCGTTGCTGTGAGGACGTT			
IL-10	Forward	GGGGCGAGTGTAACAAGACC	109	60.27	XM_036162094.1
	Reverse	GCAGAGGAGGTCACACCATTT			
TNF-α	Forward	CCCTCACACTCACAAACCAC	211	59.82	NM_001278601.1
	Reverse	ATAGCAAATCGGCTGACGGT			
Ocln	Forward	CCTCCACCCCCATCTGACTA	79	60.03	NM_001360536.1
	Reverse	GCTTGCCATTCACTTTGCCA			
ZO-1	Forward	CCTGACGGTTGGTCTTTTGC	114	59.69	NM_001163574.1
	Reverse	ACAGTTGGCTCCAACAAGGT			
β-actin	Forward	TTCGCGGGCGACGAT	297	58.57	NM_0077393.5
	Reverse	CATCTTTTCACGGTTGGCCT			

### Quantification of Small Intestine and Cecum Microbial Content in Mice

At the end of the experimental period, eight mice from each group were sacrificed humanly and then, small intestine and cecum contents were aseptically collected into sterile tubes and stored at –80°C for further analysis. An aliquot of fresh small intestine and cecum contents (1 g) were diluted in sterile saline solution at a ratio of 1:10. Diluted contents were vortexed for 2 min to obtain a homogeneous suspension, thereafter, 0.1 mL was spread on different culture media for total bacterial count. CHROMagar agar (Qingdao Hope Bio-Technology Co., Ltd., Qingdao, China) was used to enumerate *E. coli*, bismuth sulfite agar (Qingdao Hope Bio-Technology Co., Ltd., Qingdao, China), for *Salmonella* enumeration, mannitol salt agar (Qingdao Hope Bio-Technology Co., Ltd., Qingdao, China) for *Staphylococcus aureus* whereas De Man Rogosa Sharpe agar (Guangzhou Ikeme Technology Co., Ltd., Guangzhou, China) was used for the growth and enumeration of *Lactobacillus* species. The plates were incubated under anaerobic conditions at 37°C for 24 h. Furthermore, colonies obtained on the plates were counted and microbial populations were expressed as log_10_ CFU/g of small intestine and cecum contents.

### Statistical Analysis

Statistical analysis of the data collected (from various independent experiments) was performed using GraphPad Prism 5.04 software (GraphPad Software, Inc., La Jolla, CA, United States) and SPSS 20 Statistical Analysis Software (SPSS Inc., Chicago, IL, United States). The Shapiro–Wilk Test was used to test the normality of the data. The experimental data were first tested by normal distribution, and on this basis, we carried out one-way analysis of variance (ANOVA), in which the statistics included the homogeneity test of variance. All experimental results are presented as mean ± SD, and statistical significance were determined by one-way analysis of variance (ANOVA) followed by the Tukey’s test. The values were significantly different at *P* < 0.05.

## Results

From the 13 distinctive bacteria colonies on the LB agar of both cultured leaves and stems of *A. adenophora*, two colonies were selected based on the results of the preliminary (colony color, gram staining, and catalase test), morphology (bacteria size, shape, and arrangements) and biochemical (NaCl tolerance) tests. The two endophytes (one from leaf and another from the stem labeled as LB60 and LB68 respectively) were gram positive, creamy white, NaCl tolerant and catalase negative which were basic characteristics for bacteria to be classified as a probiotic (Alfonzo et al., 2016), hence were selected for further probiotic test (pH, and bile salt tolerance, antibiotic sensitivity, antimicrobial activity, and sugar fermentation), identified (using 16S rRNA) and used for the animal experiment.

### Morphological and Biochemical Characterization

The morphological characteristics of the selected endophytes (LB60 and LB68) are shown in Table 3.

**TABLE 3:** Preliminary, morphological, and biochemical characteristics of LB60 and LB68 bacteria isolated from *Ageratina adenophora.*

	Parameters	LB68	LB60
	Part of plant isolated	Stem	Leaf
	Size	1 mm	2 mm
Characteristics	Culture media characteristics	Colonies round, creamy, smooth, convex, moist, raised and entire	Colonies irregular, creamy, mucoid, opaque, raised and wavy
	Isolates morphology	Singles rods	Rods in chains (2–3 pairs)
	Gram staining	Positive (+)	Positive (+)
	Catalase	Negative (–)	Negative (–)
	Glucose	Positive (+)	Positive (+)
Sugar	Sucrose	Positive (+)	Positive (+)
fermentation	D-sorbitol	Negative (–)	Positive (+)
	2.5	++	++
Varying pH	4.5	++	++
	6.5	++	++
	0	++	++
Bile salt	0.2	++	++
concentration	0.4	++	++
(%)	0	++	++
NaCl	2	++	++
concentration	4	++	++
(%)	6.5	++	++
	Streptomycin (10 μg)	12^MS^	22^S^
	Tetracycline (30 μg)	21^S^	28^S^
Antibiotics	Erythromycin (15 μg)	–^R^	8^R^
	Ampicillin (20 μg)	–^R^	–^R^
	Sultamicillin (10 μg)	30^S^	25^S^
	*E. coli* CMCC44103	–	–
Antibacterial	*Salmonella typhimurium* S19	–	–

***++,** Turbidity of the growth medium. Resistant (R) (zone < 10 mm), moderately susceptible (MS) (zone 10–20 mm) and susceptible (S) (zone > 20 mm) and very susceptible (VS) (zone > 31 mm). “**–**” No zone of inhibition on growth medium.*

The results of the catalase test are listed in Table 3. Both LB60 and LB68 endophytes produced no bubbles with 3% H_2_O_2_ (catalase negative).

In this study, a change in the culture medium from red to yellow gave an indication of acid production during carbohydrate fermentation test. LB68 could change the color of the medium for both glucose and sucrose but not D-sorbitol; however, LB60 changed the medium for all tested sugars (Table 3).

Furthermore, the NaCl tolerance test after 24 h showed that, both endophytes (LB60 and LB68) showed growth in broths of all tested NaCl concentrations (Table 3).

### Probiotic Characteristics Test

From the results, both endophytes (LB60 and LB68) showed growth in all the broths at different pH and bile salt concentrations (Table 3). Moreover, the antibiotic resistance test revealed that both LB60 and LB68 were susceptible to streptomycin, tetracycline and sultamicillin but resistant to erythromycin and ampicillin (Table 3). In addition, results from the antimicrobial test showed no zone of inhibition on agar of the tested pathogenic bacteria for either endophyte (Table 3).

#### Identification of LB60 and LB68

**FIGURE 1:**
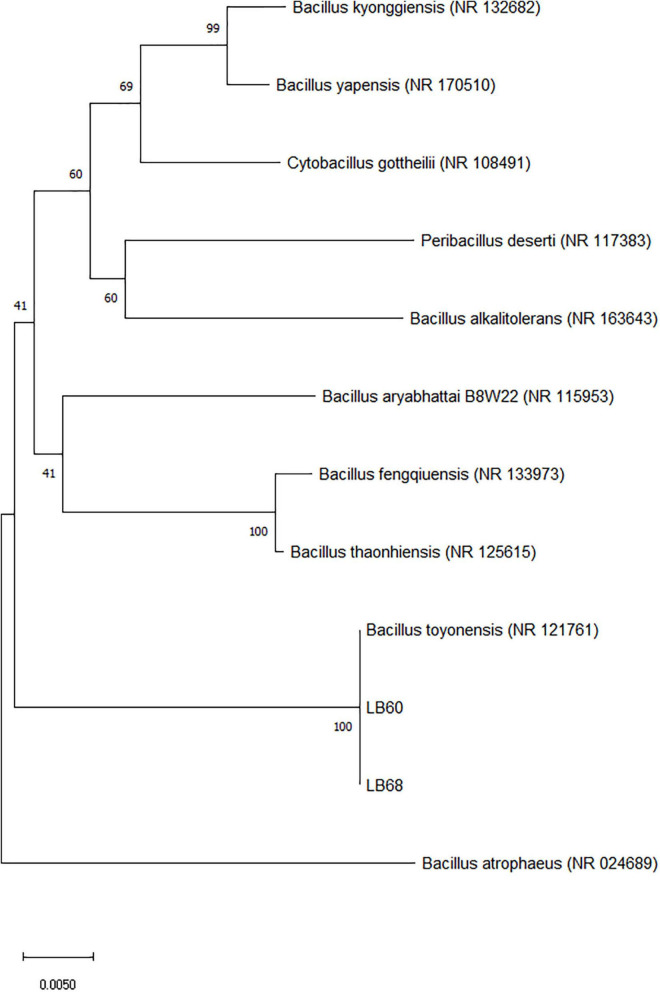
Phylogenetic analysis of the isolates (filled rectangle) based on the 16S rRNA gene sequences. Accession numbers for the available 16S rRNA gene sequences used are given in parentheses behind species and strain names. The phylogenetic tree was calculated and drawn by using the MEGA version X software after multiple alignments of the data by CLUSTAL W. The tree was constructed using the neighbor-joining method, and the evolutionary distances were computed using the p-distance method. The numbers at the branches are bootstrap confidence percentages (%) based on 1000 resampled data sets. Bar, 0.0050 substitutions per nucleotide position.

Two strains, LB60 and LB68, were screened from 13 colonies for further evaluation based on their performance on LB agar plates. In the assay, LB60 and LB68 showed the largest clearing zones; isolation was carried out based on the appearance of LAB and the clearing zone around the colony on LB agar plates. Under the microscope, bacteria were characterized as rod-shaped and gram-positive. For catalase activity, all the strains were negative (no bubbles were observed). Based on the results of 16S rRNA sequencing, LB60 had high sequence identity (99.86%) to *Bacillus toyonensis* strain BCT-7112, and LB68 had high sequence identity (99.93%) to *Bacillus toyonensis* strain BCT-7112. A phylogenetic tree was constructed (Figure 1) using the 16S rRNA sequences, which demonstrated that, the selected strains belonged to *Bacillus toyonensis*. The BLAST result of the sequence were submitted to Genbank (accession numbers: MW287198 and MW287199 (LB60 and LB68 respectively). The new strains were named as *Bacillus toyonensis* SAU-19 (LB60) and *Bacillus toyonensi*s SAU-20 (LB68) respectively.

### Effects of *Bacillus toyonensis* SAU-19 and SAU-20 on Feed Intake and Weight of in Mice Fed High Fat Diet

Based on our observations all the treatment groups appeared healthy and active. No deaths or signs of gastrointestinal upset like diarrhea occurred in the treatment groups (data not shown). In addition, *Bacillus toyonensis* SAU-19 and SAU-20 groups decreased the weight associated with high fat diet (*P* < 0.05) as compared to the HF group (Table 4) after the 35 days’ experimental period. However, there was no difference between the *Bacillus toyonensis (B. toyo.)* groups and the LGG group (*P* > 0.05) in terms of weight gain. Weight gain in the *B. toyo.* SAU-19 group were also not significantly different from those in the *B. toyo.* SAU-20 group. Therefore, these results indicated that *B. toyo.* SAU-19 and *B. toyo.* SAU-20 could improve growth and immunity just as commercial probiotic (LGG), hence can be used in treating obesity induced by high fat diets.

**TABLE 4:** Effect of *Bacillus toyonensis* SAU-19 and SAU-20 on Average daily feed intake (g/d/mice) and Average weight (g/mice) of mice fed high fat diet.

	**Treatments**				
**Growth parameters**	**NC**	**HF**	**HF + LGG**	**HF + *B. toyo*. SAU-19**	**HF + *B. toyo.* SAU-20**
Average Daily Feed intake^1^ (g/d/mice)	5.00 ± 0.002	4.99 ± 0.002	4.99 ± 0.02	4.99 ± 0.002	5.00 ± 0.002
Initial Average body weight (g/mice)	26.92 ± 0.84	26.49 ± 0.17	26.41 ± 0.14	26.97 ± 0.24	26.80 ± 0.06
Final Average body weight^2^ (g/mice)	33.48 ± 0.41^b^	37.82 ± 1.13^a^	34.83 ± 0.28^b^	35. 05 ± 0.55^b^	35.16 ± 1.39^b^

*The table is represented as means value ± standard deviation (SD). Columns with different superscripts ^a,b^ are statistically different (n = 12, P < 0.05).*

*NS, mice were administered normal feed and normal saline; HF, mice were fed high fat diet and normal saline; HF + LGG, mice were administered high fat diet and 10^6^ Lactobacillus rhamnosus GG; HF + B. toyo. SAU-19, mice were administered with high fat diet and 10^6^ Bacillus toyonesis SAU-19 from A. adenophora; HF + B. toyo. SAU-20, mice were administrated with high fat diet and 10^6^ Bacillus toyonensis SAU-20 from A. adenophora.*

*^1^Average daily feed intake = Weight of feed given (g/day/mice)-Weight of feed remaining (g/day/mice).*

*^2^Average body weight = Individual weights/total number of mice.*

### Histopathological Results

**FIGURE 2:**
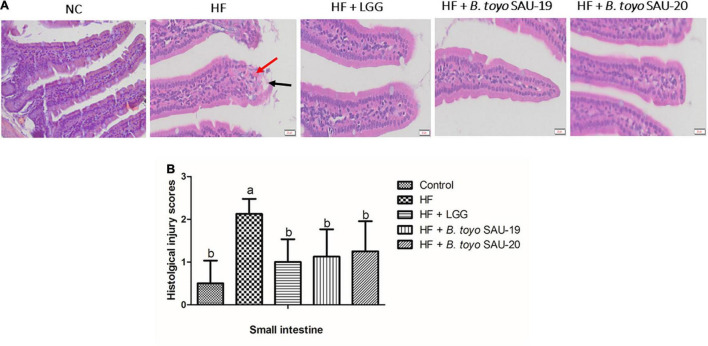
Histopathological observation of in the small intestinal sections of mice fed *Bacillus toyonensis* SAU-19 and SAU-20 after 35 days. (A) Photograph of histopathological staining in treatment groups (Scale = 20 μm). (B) Histological inflammation damage scores of ileum section of the small intestine of mice. The values are presented as the mean ± SD. Bars without the same letter differed significantly (*n* = 4, *P* < 0.05). The HF group showed moderate pathological changes characterized by bleed, and moderate inflammation, however, these were mild in the treatment groups. Bleeds are indicated by red arrow and structural damage are indicated by black arrow. NC, mice were administered normal diet + normal saline; HF, mice were fed high fat diet + normal saline; HF + LGG, mice were administered high fat diet + 10^6^ *Lactobacillus rhamnosus* GG; HF + *B. toyo.* SAU- 19, mice were administered high fat diet + 10^6^ *Bacillus toyonensis* SAU-19 from *A. adenophora*; HF + *B. toyo.* SAU-20, mice were administrated with high fat diet + 10^6^ *Bacillus toyonensis* SAU-20 from *A. adenophora*.

As shown in Figure 2A, the structure of the small intestine of the control and treatment groups were complete and showed mild pathological changes compared to the HF group. The sub-mucosa, muscle layer and intestinal gland were arranged normally, and the morphology of the lamina propria and epithelial layer were complete. In addition, the mucosa and muscle layer in the treatment groups were clear compared to those in the HF group. The HF group showed moderate pathological changes characterized by intestinal structure damage, bleeding, and mild local inflammatory infiltration of the epithelial layers. Moreover, histological examination of ileum sections showed no significance difference in the cumulative injury scores of the treatment groups compared with the control group (Figure 2B, *P* < 0.05). The injury score for the HF group was higher that the control and the treatment groups (*P* < 0.05). Furthermore, there was no difference in the structure and injury scores among *B. toyo.* SAU-19, *B. toyo. SAU*-20 and *LGG* groups. These results indicated that, *B. toyo.* SAU-19 and *B. toyo.* SAU-20 could improve the structure of intestine from high fat diet induced injury.

### Effects of *Bacillus toyonensis* SAU-19 and SAU-20 on Villi Height and Crypt Depth of the Small Intestine in Mice Fed High Fat Diet

**FIGURE 3:**
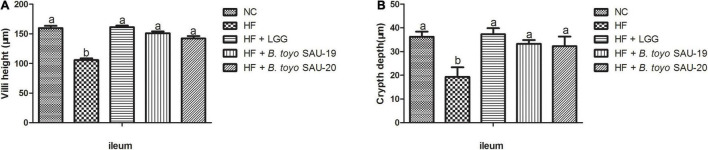
Effects of *Bacillus toyonensis* SAU-19 and SAU-20 on the villi height and crypt depth of small intestine in mice fed high fat diet. (A) Villi height of ileum in mice. (B) Crypt depth of ileum in mice. The values are presented as the mean ± SD. Bars without the same letter differed significantly (*n* = 4, *P* < 0.05). NC, mice were administered normal diet + normal saline; HF, mice were fed high fat diet + normal saline; HF + LGG, mice were administered high fat diet + 10^6^ *Lactobacillus rhamnosus* GG; HF + *B. toyo.* SAU-19, mice were administered high fat diet + 10^6^ *Bacillus toyonensis* SAU-19 from *A. adenophora*; HF + *B. toyo.* SAU-20, mice were administrated with high fat diet + 10^6^ *Bacillus toyonensis* SAU-20 from *A. adenophora*.

The villi height (V) and crypt depth (C) of the ileum section of the small intestine of the high fat diet group (HF) was lower that the control whereas the V and C of the treatment groups were significantly higher than that of the HF group (Figure 3, *P* < 0.05). Moreover, there was no difference in villi height and crypt depth of the ileum section among the treatment groups (*P* > 0.05). Therefore, we stipulated that, *B. toyo.* SAU-19 and *B. toyo.* SAU-20 protected or maintained the ileum structure from high fat diet destruction just as the commercial probiotic (LGG).

### Effects of *Bacillus toyonensis* SAU-19 and SAU-20 on Antioxidant Indexes and DAO Activity in Mice Fed High Fat Diet

**FIGURE 4:**
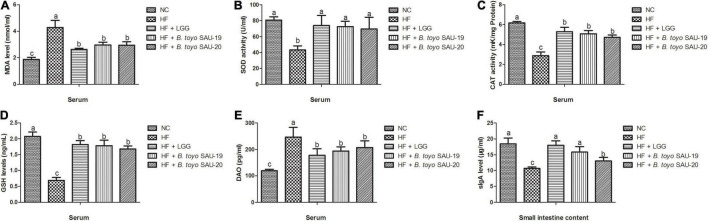
Effect of *Bacillus toyonensis* SAU-19 and SAU-20 on antioxidant indexes, DAO and sIgA activity in mice fed high fat diet (A) levels of MDA enzyme in serum, (B) levels of SOD enzyme in serum, (C) levels of CAT enzyme in serum (D) levels of GSH enzyme in serum (E) DAO concentrations in the serum, (F) sIgA levels in small intestine contents. Bars represent means ± standard deviation (SD). The values are presented as the mean ± SD. Bars without the same letter differed significantly (*n* = 6, *P* < 0.05). NC, mice were administered normal diet + normal saline; HF, mice were fed high fat diet + normal saline; HF + LGG, mice were administered high fat diet + 10^6^ *Lactobacillus rhamnosus* GG; HF + *B. toyo.* SAU-19, mice were administered high fat diet + 10^6^ *Bacillus toyonensis* SAU-19 from *A. adenophora*; HF + *B. toyo.* SAU-20, mice were administrated with high fat diet + 10^6^ *Bacillus toyonensis* SAU-20 from *A. adenophora*.

The results of this study showed that, all the treatment groups and control had reduced serum MDA levels compared to the HF group (Figure 4A, *P* < 0.05). In contrast, SOD, CAT, and GSH levels in the treatment and control groups were significantly higher than those in the HF group (Figures 4B–D, *P* < 0.05). However, we observed that the levels of MDA in the treatment groups were higher than the control (NC), whereas the levels of CAT and GSH in the treatment group lower than that of the control group (*P* < 0.05). There was no significance difference in SOD, CAT, and GSH levels among the LGG, *B. toyo.* SAU-19 and *B. toyo.* SAU-20 treatment groups (*P* > 0.05).

From the results, we also observed that, the levels of DAO in the treatment groups were lower than those in the HF group, however, the DAO levels of the treatment groups were significantly higher than the control group (Figure 4E, *p* < 0.05). We also observed no difference among the NC, LGG, *B. toyo.* SAU-19 and *B. toyo.* SAU-20 groups (*P* > 0.05). These results indicate that the two isolated strains improved the barrier integrity of the intestine.

### Effects of *Bacillus toyonensis* SAU-19 and SAU-20 on sIgA Levels in the Small Intestine Contents of Mice Fed High Fat Diet

For the sIgA levels in the small intestine contents, we observed that, the sIgA levels in the treatment groups were higher compared to the HF group (Figure 4F, *P* < 0.05). We also observed that, the sIgA levels in the control (NC) and LGG group were higher than those in the *B. toyo.* SAU-20 (*P* < 0.05). However, no differences were observed among the NC, LGG and the *B. toyo.* SAU-19 groups (*P* > 0.05).

### Effects of *Bacillus toyonensis* SAU-19 and SAU-20 on Tight Junction Proteins in the Small Intestine Tissues of Mice Fed High Fat Diet

**FIGURE 5:**
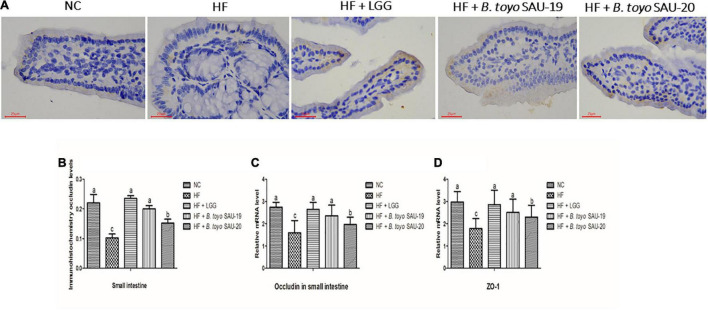
Effects of *Bacillus toyonensis* SAU-19 and SAU-20 on tight junction proteins of the small intestine tissue in mice fed high fat diet. (A) Immunohistochemistry staining photographs of intestinal sections (yellow area represents occludin). (B) Immunohistochemical analyses of occludin levels in small intestine of mice in each group Bars represent means ± standard deviation (SD), the values are presented as the mean ± SD. Bars without the same letter differed significantly (*n* = 4, *P* < 0.05). (C) mRNA expression of occludin in small intestine tissues (D) mRNA expression of ZO-1 in small intestine tissues. Bars represent means ± standard deviation (SD) for eight mice per treatment. The values are presented as the mean ± SD. Bars without the same letter differed significantly (*p* < 0.05). NC, mice were administered normal diet + normal saline; HF, mice were fed high fat diet + normal saline; HF + LGG, mice were administered high fat diet + 10^6^ *Lactobacillus rhamnosus* GG; HF + *B. toyo.* SAU-19, mice were administered high fat diet + 10^6^ *Bacillus toyonensis* SAU-19 from *A. adenophora*; HF + *B. toyo.* SAU-20, mice were administrated with high fat diet + 10^6^ *Bacillus toyonensis* SAU-20 from *A. adenophora*.

The immune-histochemical analyses showed that, the occludin levels in treatment and control groups were increased as compared to the HF group (Figures 5A,B, *P* < 0.05). Furthermore, we observed that, the level of occuldin in the LGG and *B. toyo.* SAU-19 groups was significantly higher than that of the *B. toyo.* SAU- 20 groups (*P* < 0.05), but no difference was observed between the LGG and the *B. toyo.* SAU-19 groups. Furthermore, the results of the relative mRNA expression of tight junction proteins (TJP) showed that, the levels of both TJPs (occludin and Z0-1) were significantly higher in the treatment groups as compared to the HF group (Figures 5C,D, *P* < 0.05). However, the LGG and *B. toyo.* SAU-19 group had an elevated expression levels of occludin and ZO-1 compared to the *B. toyo.* SAU-20 group (*P* < 0.05). No difference existed between the LGG and the *B. toyo.* SAU-19 groups.

### Effects of *Bacillus toyonensis* SAU-19 and SAU-20 on Relative mRNA Expressions Pro- and Anti-inflammation Related Cytokines in the Small Intestine Tissues of Mice Fed High Fat Diet

**FIGURE 6:**
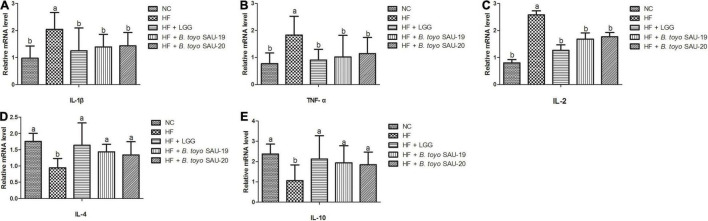
Effects of *Bacillus toyonensis* SAU-19 and SAU-20 on relative mRNA expressions of pro- and anti-inflammation related cytokines in the small intestine tissue of mice fed high fat diet (A–C) mRNA expressions of pro-inflammatory cytokines (D,E) mRNA expressions of anti-inflammatory cytokines. Bars represent means ± standard deviation (SD). The values are presented as the mean ± SD. Bars without the same letter differed significantly (*n* = 6, *P* < 0.05). NC, mice were administered normal diet + normal saline; HF, mice were fed high fat diet + normal saline; HF + LGG, mice were administered high fat diet + 10^6^ *Lactobacillus rhamnosus* GG; HF + *B. toyo.* SAU-19, mice were administered high fat diet + 10^6^ *Bacillus toyonensis* SAU-19 from *A. adenophora*; HF + *B. toyo.* SAU-20, mice were administrated with high fat diet + 10^6^ *Bacillus toyonensis* SAU-20 from *A. adenophora*.

The results of the relative mRNA expression of inflammation related cytokines showed that, the treatment groups decreased the levels of pro-inflammatory cytokines (IL-1β, TFN-α, IL-2) and increased the level of the anti-inflammatory cytokines (IL-4 and IL-10) in small intestine tissues as compared to the HF group (Figures 6A–E, *P* < 0.05). Moreover, there was no difference in both pro- and anti-inflammatory cytokines among the *B. toyo.* SAU-19, *B. toyo.* SAU-20 and LGG groups (*P* > 0.05).

### Effects of *Bacillus toyonensis* SAU-19 and SAU-20 on Pathogenic (*Escherichia coli*, *Salmonella*, and *Staphylococcus aureus*) and Beneficial (*Lactobacillus* spp.) Bacteria Enumeration in Small Intestine and Cecum Contents in Mice Fed High Fat Diet

**FIGURE 7:**
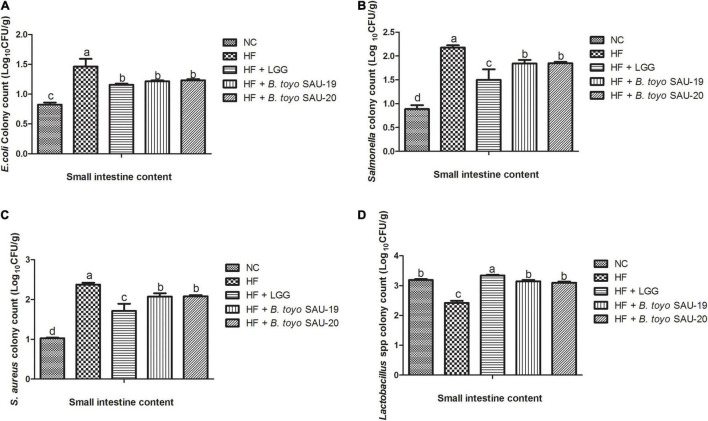
Effects of *Bacillus toyonensis* SAU-19 and SAU-20 on bacteria enumeration in small intestine contents of mice fed high fat diet (A) colony count of *Escherichia coli*, (B) colony count of *Salmonella* spp., (C) colony count of *Staphylococcus aureus*, (D) colony count of *Lactobacillus* spp. The values are presented as the mean ± SD. Bars without the same letter differed significantly (*n* = 6, *P* < 0.05) the same. NC, mice were administered normal diet + normal saline; HF, mice were fed high fat diet + normal saline; HF + LGG, mice were administered high fat diet + 10^6^ *Lactobacillus rhamnosus* GG; HF + *B. toyo.* SAU-19, mice were administered high fat diet + 10^6^ *Bacillus toyonensis* SAU-19 from *A. adenophora*; HF + *B. toyo.* SAU-20, mice were administrated with high fat diet + 10^6^ *Bacillus toyonensis* SAU-20 from *A. adenophora*.

**FIGURE 8:**
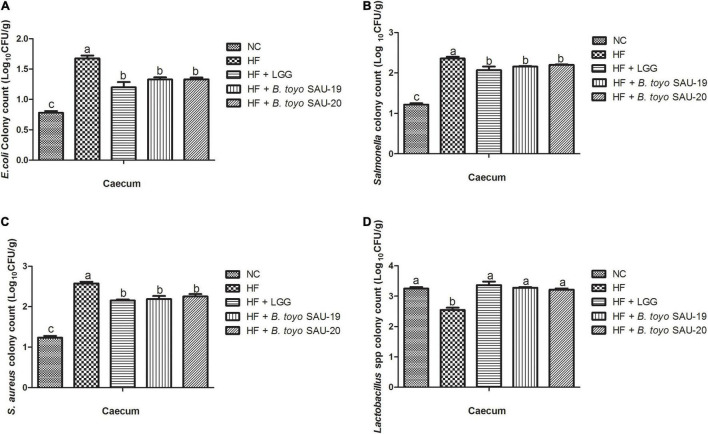
Effects of *Bacillus toyonensis* SAU-19 and SAU-20 on bacteria enumeration in cecum contents of mice fed high fat diet (A) colony count of *Escherichia coli*, (B) colony count of *Salmonella* spp., (C) colony count of *Staphylococcus aureus*, (D) colony count of *Lactobacillus* spp. Bars represent means ± standard deviation (SD). The values are presented as the mean ± SD. Bars without the same letter differed significantly (*n* = 6, *P* < 0.05) the same. NC, mice were administered normal diet + normal saline; HF, mice were fed high fat diet + normal saline; HF + LGG, mice were administered high fat diet + 10^6^ *Lactobacillus rhamnosus* GG; HF + *B. toyo.* SAU-19, mice were administered high fat diet + 10^6^ *Bacillus toyonensis* SAU-19 from *A. adenophora*; HF + *B. toyo.* SAU-20, mice were administrated with high fat diet + 10^6^ *Bacillus toyonensis* SAU-20 from *A. adenophora*.

The results of the microbial count of the small intestine and cecum contents are shown in Figures 7, 8. These results showed that, the *E. coli*, *Salmonella* spp., and *Staphylococcus aureus* (*S. aureus*) counts (population) in the small intestine contents was significantly lower in the Control, LGG, *B. toyo.* SAU-19 and *B. toyo.* SAU-20 treatment groups as compared to the HF group (Figures 7A–C, *P* < 0.05). Moreover, both *B. toyo.* SAU-19 and *B. toyo.* SAU-20 treatment groups increased the number of *Lactobacillus* spp bacteria in the small intestine contents as compared to the HF (Figure 7D, *P* < 0.05).

Similarly, the count of *E. coli*, *salmonella*, and *S. aureus* in the cecum content were significantly lower in the Control, *B. toyo.* SAU-19 and *B. toyo.* SAU-20 treatment groups compared to the HF group (Figures 8A–C, *P* < 0.05). Finally, the *B. toyo.* SAU-19 and *B. toyo.* SAU-20 treatment groups increased the number of *Lactobacillus* spp. bacteria in the cecum as compared to the HF group (Figure 8D, *P* < 0.05). Moreover, there was no difference in bacteria enumeration between *B. toyo.* SAU-19 and *B. toyo.* SAU-20. We further observed that the counts of *Salmonella* and *S. aureus* in the small intestine content of the LGG group was lower compared to the *B. toyo.* SAU-19 and *B. toyo.* SAU-20 (*P* < 0.05). In addition, the *Lactobacillus* spp. count in the small intestine of the LGG group was higher than that of *B. toyo.* SAU-19 and *B. toyo.* SAU-20 (*P* < 0.05).

## Discussion

*Bacillus toyonensis* is a naturally occurring, non-toxigenic and non-pathogenic strain of *B. cereus*, and which does not cause any adverse effects in animals such as rabbits, pigs, chickens, turkeys and cattle (Jimenez et al., 2013; EFSA, 2014). Many studies have revealed probiotic activities of the bacteria (Taras et al., 2005). *Bacillus toyonensis* have been reported to increase weight gain, improved feed conversion ratios, reduce post-weaning diarrhea and lower mortality rates of piglets (Baum et al., 2002). The results of our 16S rRNA and the phylogenetic tree demonstrated that, the selected probiotic endophyte strains were similar to *Bacillus toyonensis* and the morphological analysis results showed that, the strains had similar morphology as the general characteristics of *Bacillus* spp. used as probiotics (Özüsağlam and Aksaray, 2010), however, differed in some colony morphological characteristics like margins, elevation and colony size as well as sugar fermentation. Therefore, we hypothesized that *Bacillus toyonensis* SAU-19 (LB60) and SAU-20 (LB68) may possess probiotic properties. Both tested endophytes successfully fermented glucose and sucrose except for D-sorbitol which was fermented only by LB60, were tolerant to NaCl, low pH and bile salt. This results were similar to the works of Nithya and Halami (2013) and Mok et al. (2020).

Moreover, the antibiotic resistance and antimicrobial activities of probiotic strains are essential to prevent or reduce infections caused by pathogenic bacteria. In this study, it was observed that, both *Bacillus toyonensi*s (LB60 and LB68) were resistant to ampicillin and erythromycin, however, they were susceptible to streptomycin, tetracycline, and sultamicillin according to the method described by Talib et al. (2019). This results were consistent with the study of Glenwright et al. (2017) and Fiedler et al. (2019). Furthermore, both *Bacillus toyonensi*s (LB60 and LB68) had no inhibitory effect on tested pathogenic bacteria, hence, we concluded that, the endophytic bacteria did not produce antimicrobial substances. This finding were similar to the study of Agamennone et al. (2019) and Rojas-Solis et al. (2020).

High fat diets has been reported to increase weight gain, affect immune organ weight and indices (Jin et al., 2017; Li et al., 2020; Yoshizaki et al., 2020). Probiotics reduce excessive weight increase in animals fed high fat diet (Kong et al., 2019; Chen Y. T. et al., 2021). From the results of the *in vivo* experiments, we observed that, *Bacillus toyonensis* SAU-19 and SAU-20 could reduce weight gain in mice compared to the HF group. Similar results were obtained from a previous study by Kantas et al. (2015), and Cai et al. (2020) which reported that, probiotics could reduce the weights associated with high fat diet in animals.

Numerous studies have reported on the association of high fat diet to the distortion of the gut microbiota and pathological changes in the intestine (Netto Candido et al., 2018). This may be as a results of increasing harmful bacteria and viruses which invade tissues by producing lethal toxins and metabolites which may cause damages in the GIT (Abaidullah et al., 2019). The pathological results obtained in this study showed that administering *Bacillus toyonensis* SAU-19 and SAU-20 did not cause any severe pathological changes in the small intestine tissues as compared to the HF group that showed a moderate pathological changes. Complications such as bleeding, and moderate inflammation were observed in the small intestines of mice in the HF group, however this was mild in the treatment groups. This was consistent with previous reports by Ringø et al. (2007) who stated that, probiotics could reverse the intestinal pathological damage caused by pathogenic bacteria.

Furthermore, villus height (V), crypt depth (C), and V:C ratio are major morphological indicators that are used to determine the general health and functions of the small intestine (Feng et al., 2016; Cui et al., 2021). Villi are important structures found in the small intestine and are mainly involved in the absorption of nutrients (Fuller, 2004). The higher the villi height and the deeper the crypt depth the higher the surface area for nutrient absorption in the small intestine (Chwen et al., 2013). In this study, it was observed that, the ileum section of the small intestine in the treatment groups had significantly higher villi heights and crypt depth as compared to the high fat diet (HF) group, indicating that, *Bacillus toyonensis* SAU-19 and SAU-20 could improve the villi structure thereby increasing its height and crypt depth. This result was consistent with a previous study by Jayaraman et al. (2013) and Wang et al. (2019) who reported that, probiotics could improve villi length and crypt depth in animals.

The intestinal barrier is a complex multilayer system, consisting of an external anatomical barrier and an inner functional immunological barrier (Zheng et al., 2019). Chemical markers (such as DAO and D-lactic acid) are important indicators of intestinal permeability. When the intestinal barrier function is damaged, these chemical makers are released into the blood and these are employed as indicators for intestinal integrity (Chen F. et al., 2021). Our results showed that, all the treatment groups could reduce the levels of DAO in mouse serum, however, only LGG and *Bacillus toyonensis* SAU-19 showed a significant reduction compared to the HF group. This is similar to other studies that reported that, probiotics reduce the level of DAO in the serum (Ding et al., 2019).

Oxidative stress is a harmful physiological reaction, which can lead to excessive re-active oxygen species (ROS) generation (Pizzino et al., 2017). Various studies have reported that; oxidative stress reduces growth performance in animals (Lv et al., 2012). MDA is an oxidative stress indicator whereas SOD, CAT and GSH-PX are main antioxidant enzymes in mammals (Wagner et al., 1994; Ighodaro and Akinloye, 2018). High fat diet has been linked with oxidative stress (Li et al., 2019; Tan and Norhaizan, 2019). Numerous studies have also reported that supplementation of exogenous probiotics has a beneficial effect on preventing oxidative stress. Our results showed that *Bacillus toyonensis* SAU-19 and SAU-20 reduced the MDA levels and increased the SOD, CAT, and GSH activity in the serum. This result is consistent with a previous study by Yau et al. (2020) which reported that, *Lactobacillus rhamnosus* GG could improve the antioxidant capacity of mice fed high fat diet.

Secretory immunoglobulin A (sIgA) is a predominant immunoglobulin in the mucosal system and is critical for protecting mucosal surfaces against toxins, viruses, and enteropathogens (Xin et al., 2020). It neutralizes or prevent pathogens from binding to the mucosal surface of the intestine (Lindner et al., 2012), thereby protecting the intestine. sIgA also plays a role in the preservation of mucosal homeostasis, which determines the intestinal microbiota composition and enhances the development of systemic immunity (Lammers et al., 2010). Furthermore, sIgA suppresses the release of pro-inflammation indicators that initiate intestinal diseases by coating a portion of commensal intestinal bacteria in humans and animals (Van et al., 2004; Palm et al., 2014; Xin et al., 2020). Our results showed that both *Bacillus toyonensis* SAU-19 and SAU-20 increased sIgA levels in the small intestine contents. This result indicated that, these two strains improved intestinal health. The results of this study are consistent with a previous study by Kabeerdoss et al. (2011) which reported that, probiotic interventions could increase the fecal IgA production in healthy children, preterm infants, and piglets.

Intestinal TJP, such as claudin, ZO, and occludin, have been reported to be directly associated with the normal function of the intestinal mucosa barrier (Wu et al., 2018). The leaking gut is often associated with reduced expression or internalization of TJPs such as occludin and ZO (Buschmann et al., 2013). High-fat diet (HFD)-feeding increases intestinal permeability by impairing tight junction (TJ) protein function, which may explain the associated pathologies (Chen K. et al., 2020). Our results showed that, both *Bacillus toyonensis* SAU-19 and SAU-20 increased the expression levels of TJ proteins (Occludin and ZO-1) in the small intestine of high fat diet fed-mice thereby conferring a beneficial effect on mucosal barrier function. This is in agreement with a previous study by Gong et al. (2016) which reported that, *Bacillus subtilis* administration could elevate the expression of TJ proteins (claudin-1, occludin, JAM-A, and ZO-1) in mice.

Furthermore, the abnormal expression of TJP is closely associated with the inflammatory responses of the intestine (Xu et al., 2016). Cytokines, as endogenous mediators of the immune system that control the occurrence of inflammatory reaction (Jiang et al., 2019). When there is acute toxicity (such as toxin metabolism), the levels of IL-6, IL-2, tumor necrosis factor and other inflammatory factors increase (Zhang et al., 2018). The intestinal barrier does not only protect the deeper layers of the intestinal wall, but also tightly regulates the passage of pro-inflammatory molecules, microorganisms, toxins, and antigens (Farré et al., 2020). High expression of pro-inflammation cytokines such as IL-1β and TNF-α, encourage intestinal epithelial cells damage via autocrine/paracrine action (Van Kaer and Olivares-Villagomez, 2018). High fat diet causes inflammation characterized by elevated levels of pro-inflammation cytokines (van der Heijden et al., 2015; Dutheil et al., 2016). Our results showed that, *Bacillus toyonensis* SAU-19 and SAU-20 could reduce the expression of pro-inflammatory cytokine and increase the expressions of the anti-inflammation cytokine in small intestine compared to the high fat diet group, thereby improving immunity. This study is in agreement with a previous study by Roos et al. (2018).

The gut microbiota is altered by feeding high fat diet (Bibbò et al., 2016; Guo et al., 2017). Exogenous probiotics improve growth performance by altering gut microbiota (Xin et al., 2020). Our current study showed that *Bacillus toyonensis* SAU-19 and SAU-20 isolated from *A. adenophora* exert beneficial effects on intestinal flora. Specifically, *Bacillus toyonensis* SAU-19 and SAU-20 markedly enhanced *Lactobacillus* spp. populations and reduced *Enterobacteriaceae* populations (*E. coli*, *Salmonella*, and *Staphylococcus aureus*) compared to the high fat diet group. Many studies have reported that, *Bacillus toyonensis* could inhibit the activities of *Salmonella enteritidis* and *E. coli* in the gut of pigs (Simon, 2010). Therefore, it was speculated that, *Bacillus toyonensis* SAU-19 and SAU-20 played a role in reducing the harmful bacteria and increasing beneficial bacteria thus, balancing the gut microbiota. However, the mechanism for achieving this activity was not from the production of antimicrobial substances and this was confirmed by the antimicrobial test in this study. Even though a study by Gonzalez-Ortiz et al. (2016) reported that, *Bacillus toyonensis* effectively reduced *E. coli* activity in the gut mainly by inhibiting the quorum sensing of the pathogenic bacteria there is still the need for an in-depth study to reveal the main molecular mechanism of these strains gut modulation activities.

In summary of the results, this study reported on the cross talk between gut microbiota and probiotics. We observed changes in the gut microbiota composition in the high fat diet fed-mice characterized by an increase in the pathogenic bacteria population. In addition, we also observed that the intestinal structure and barrier was damaged in the high fat diet fed-mice. However, we did not observe these changes in the normal mice. Therefore, we concluded that, the impairment of the gut structure and integrity was as a result of dysbiosis of the gut microbiota. Furthermore, the administration of SAU-19 and SAU-20 did not cause any obvious damage to the intestine or change the gut microbiota composition compared to the normal mice, hence we concluded that these two strains protected or prevented the intestine from damages associated with gut dysbiosis in mice fed high fat diet.

Even though this study has revealed the probiotic potential of the *B. toyonensis* SAU-19 and SAU-20, this study did not investigate the main mode of action of these bacteria strains in reducing the population of the pathogenic bacteria in the gut. In addition, we only quantified the limited small intestine and cecum microbial contents using the plate count method. Moreover, this study also lacked the correlation analysis of the gut microbiota and their metabolites function in improving the gut health. Thus, there is the need for further research to ascertain the exact molecular mechanisms of these two strains in maintain the gut structure and integrity as well as performing microbial diversity analysis based on next-generation sequencing to clearly reveal the complete changes and correlation of the gut microbiota and their metabolites as a results of *Bacillus toyonensis* SAU-19 and SAU-20 administration.

## Conclusion

We performed a preliminary study and isolated and identified two potential probiotic bacteria endophytes (*Bacillus toyonensis* SAU-19 and SAU- 20) from *A. adenophora*. Both *Bacillus toyonensis* strains prevented the excessive increase in body weights associated with high fat diet feeding, improved the antioxidant status, alleviated structure and integrity damage of the small intestine and reduced the levels of pathogenic bacteria (*E. coli, Salmonella*, and *Staphylococcus aureus*) without causing any health complications in mice fed high fat diet. Therefore, *Bacillus toyonensis* SAU-19 and SAU-20 may be used as future probiotics in humans and animals. However, further studies are still needed to clarify the detailed mechanisms of action by validating the efficacy of *B. toyonensis* SAU-19 and SAU-20 through human clinical trials.

## Data Availability Statement

Publicly available datasets were analyzed in this study. This data can be found here: MW287198 and MW287199.

## Ethics Statement

The animal study was reviewed and approved by Institutional Animal Care and Use Committee of Sichuan Agricultural University, Sichuan, China, under the permit number DKY-B2019603005.

## Author Contributions

SO, JW, YC, and LX: conceptualization, methodology, and software. SO, LX, JW, SW, YR, PG, and MZ: data collection, writing, and original draft preparation. SO, YC, ZR, and YH: validation and investigation. JW, ZR, and YH: funding and supervision. All authors read and agreed to the published version of the manuscript.

## Conflict of Interest

YH was employed by New Ruipeng Pet Healthcare Group Co., Ltd. The remaining authors declare that the research was conducted in the absence of any commercial or financial relationships that could be construed as a potential conflict of interest.

## Publisher’s Note

All claims expressed in this article are solely those of the authors and do not necessarily represent those of their affiliated organizations, or those of the publisher, the editors and the reviewers. Any product that may be evaluated in this article, or claim that may be made by its manufacturer, is not guaranteed or endorsed by the publisher.

## Footnotes

1
http://www.nc3rs.org.uk/arrive-guidelines
